# Experimental, Theoretical, Numerical and Big-Data-Based Investigations on Characterizations for Geomaterials

**DOI:** 10.3390/ma16041727

**Published:** 2023-02-20

**Authors:** Shaofeng Wang

**Affiliations:** School of Resources and Safety Engineering, Central South University, Changsha 410083, China; sf.wang@csu.edu.cn

Rock and rock-like materials such as concrete, soil, and underground backfilling materials are considered to be geomaterials. Geomaterials are essential for life and engineering because they are important for human construct extraction, mining, storage, and transport areas in the Earth’s crust. Essential material that has to be faced in mining processes is rock or geomaterials. Drilling, blasting, cutting, crushing and rock support processes in geotechnical, mining, petroleum, natural gas and geothermal engineering rely largely on the physical and mechanical properties of rock. Additionally, the quarrying and machining processes of natural stone depends in a greater extent on the physical and mechanical properties of natural rock materials. In mineral processing operations, physical and mechanical properties of ore rock are important parameters at various stages such as grinding, milling, and gathering. Soil and concrete are also important geomaterials. Soil behaviour can determine the long-term stabilities of civil and geotechnical engineering. Concrete, when used together with the rock structures in excavation and backfilling operations, as support or lining construct for surround rock, can significantly affect the behaviour of the rock structure. Drilling and excavations of underground openings in the Earth’s crust are requirements for the exploitation and utilization of mineral resources, energy resources, and underground spaces. The deepest drilling depth has exceeded 12 km, and the deepest underground excavation now operates mines with depths exceeding 4 km to 5 km. Drilling, excavation, and rock support processes are essential operations for forming openings in soil or rock, which largely rely on the associated properties of geomaterials and can significantly influence the safety and stability of underground engineering. Rock excavations are faced with some instability phenomena, such as caving, rockburst, slabbing, large deformation, and zonal disintegration, posing a serious threat to the safety of mining and tunneling operations. Rock drilling also encounters many challenges deep underground. Deformation, fracture, failure, and fragmentation are the different stages of geomaterial behaviour, the monitoring and control of which are essential for ensuring drilling and excavation safety and support stability. Therefore, understanding the response processes of geomaterials during drilling and excavation activities for exploitations of georesources and geoenergy depends on the precise characterisations for geomaterials. In addition, stress conditions in geomaterials are typically characterised by combined static and dynamic loads, consisting of the excavation-induced high stress concentration around openings and strong dynamic disturbances from drilling and blasting, excavation unloading, caving, and fault slippage. It is well-recognized that geomaterial has multi-scale structures, from minerals, particles, fractures, fissures, joints, and stratification, to fault, and involves multi-scale fracture processes. Meanwhile, there are many multi-physics coupling processes in geomaterial, such as the coupled thermo–hydromechanical–chemical interaction in porous geomaterials. However, it remains a significant challenge to fully understand the deformation/fracture/failure/fragmentation behaviours and mechanisms of geomaterials in complex underground environments with complex stress conditions, multi-physics processes, and multi-scale changes.

This Special Issue aims to call for research papers and review articles encompassing in situ observations, laboratory experiments, theoretical analyses, numerical simulations, and big-data-based analyses concerning characterisations for geomaterials.

Potential topics include, but are not limited to, the following:

(1) Characterisations of geomaterials;

(2) Drilling and blasting performances of rock and rock-like materials;

(3) Cuttabilities of geomaterials;

(4) Fracabilities of geomaterials;

(5) Permeability properties of geomaterials;

(6) Dynamic response of geomaterial;

(7) Geomaterial behaviours;

(8) Geomaterial strength;

(9) Geomaterial deformation;

(10) Geomaterial fracture;

(11) Stress wave propagation in geomaterials;

(12) Thermodynamic properties of geomaterials;

(13) Beneficial utilisations of geomaterials.

Potential methodologies for studying geomaterials include, but are not limited to, the following:

(1) Experimental investigation;

(2) Field observation and monitoring;

(3) Theoretical analyses;

(4) Geostatistics;

(5) Numerical simulation;

(6) Big-data-based methods;

(7) Data mining and deep learning.

The guest editors hope that the selected papers for this Special Issue will help scholars and researchers in pushing forward and progressing in experimental, theoretical, numerical, and big-data-based investigations on characterisations for geomaterials, and provide some valuable information and recommendations for geotechnical, mining, tunnelling, and drilling engineers.

